# Printed Capillary Microfluidic Devices and Their Application in Biosensing

**DOI:** 10.3390/mi14112059

**Published:** 2023-11-04

**Authors:** Zhiyi Zhang, Stephen Lang, Kate Pearson, Yawar Farhan, Ye Tao, Gaozhi Xiao

**Affiliations:** Advanced Electronic and Photonic Research Center, National Research Council Canada, Ottawa, ON K1A 0R6, Canadakate.pearson@nrc-cnrc.gc.ca (K.P.); ye.tao@nrc-cnrc.gc.ca (Y.T.);

**Keywords:** microfluidic devices, porous materials, printing, capillary-driven flow, biosensing

## Abstract

Microfluidic devices with a free-standing structure were printed directly on polymer films using the functional materials that form interconnected pores. The printed devices can transport fluids by capillary action in the same fashion as paper-based microfluidic devices, and they can handle much smaller sample volumes than typical paper-based devices. Detection of glucose was performed using both colorimetric and electrochemical methods, and the observed limits of detection (LOD) were similar to those obtained with paper-based microfluidic devices under comparable testing conditions. It is demonstrated that printed microfluidic devices can be fabricated using printing processes that are suitable for high-volume and low-cost production and that the integration of microfluidic channels with electrodes is straightforward with printing. Several materials that are printable and form interconnected pores are presented.

## 1. Introduction

Paper-based microfluidic devices have emerged as a promising device platform for diagnostic applications since their reinvention [1,2,3]. Analytes such as metabolites, electrolytes, and enzymes have been quantified in various applications, notably, in hematology [4]. The main inherent advantage of paper-based devices lies in capillary flow, which is caused by hydrophilic cellulose fibers and the interconnected micrometer-sized pores found in paper. There is no need for external pumping [5]. Therefore, low-cost and disposable devices can be fabricated using this capillary-based platform.

Paper-based microfluidic devices are mostly fabricated by creating patterned hydrophobic barriers in hydrophilic paper, thus limiting fluid flow to within a targeted area. Filling or coating some of the pores among the hydrophilic cellulose fibers in paper using a hydrophobic material, such as wax [6,7], polydimethylsiloxane (PDMS) [8], polystyrene [9], poly(o-nitrobenzyl methacrylate) [10], is a simple way to achieve this goal. Inkjet printing, flexo printing, screen printing, plotting, stamping, chemical vapor deposition, photolithography, and other methods have been used to deposit hydrophobic materials onto paper, creating a designed pattern [5]. As the surface energy of cellulose fibers is much higher than that of hydrophobic materials, deposited solutions easily wet the fibers and penetrate the paper through the whole extent of the untreated pores. Alternatively, it is also possible to initially make paper hydrophobic using a chemical coating and then selectively activate a certain area using ion etching or chemical etching to increase the surface energy [11]. The microfluidic channels fabricated with this type of method have their side surfaces surrounded by a solid hydrophobic barrier, while their upper and lower surfaces present no barrier to water. The exposed surfaces can be protected or sealed with polymer films as required.

Paper-based microfluidic devices are also widely fabricated by simply cutting the paper. Computer-controlled blades, lasers, and other tools can be used to directly cut hydrophilic paper into a network of channels [12,13,14], allowing fluid flow in the cut-out channels. Depending on the cutting technology, polymer films can be used to support the paper from below and protect the paper’s top surface from damage during cutting. The microfluidic channels obtained by cutting have all their surfaces exposed, are difficult to handle, and need mechanical support. This is usually provided by attaching polymer films either before or after cutting [15]. The supporting polymer layer can also seal and protect the transport medium before use.

While paper-based microfluidic devices are almost exclusively fabricated with hydrophilic paper, the capability of driving fluidic flow and transporting analytes using capillary action should not be limited to paper. In principle, any material with small interconnected and hydrophilic pores should be capable of driving fluid flow by capillary action. It is, in principle, possible to fabricate new microfluidic devices with similar performance to paper-based microfluidic devices using any convenient porous material. With this understanding, specialty porous materials with hydrophilic pore walls were developed to directly print free-standing microfluidic devices on polymer films [16]. The devices produced have comparable performance to paper-based microfluidic devices, but they can be fabricated with a low-cost process and handle much smaller sample volumes. In this paper, the concept of fabricating microfluidic devices using such functional materials is realized, and the practical utility of the printed devices is demonstrated by application to glucose sensing.

## 2. Materials and Methods

### 2.1. Chemicals

Dimethyl sulfoxide (DMSO), polyvinyl alcohol (PVA) (MW 145000), fumed silica (0.007 μm), hydroxypropyl methylcellulose solution, glucose oxidase (from Aspergillus niger, 147 U mg^−1^) (GOx), horseradish peroxidase (89 U mg^−1^) (HRP), potassium iodide (KI), D-(+)-trehalose, sodium phosphate dibasic heptahydrate, sodium phosphate monobasic monohydrate, 3-Aminopropyltrimethoxysilane, dextrose, uric acid (UA), sodium citrate tribasic, creatinine, potassium chloride, and calcium chloride were purchased from Millipore Sigma (Oakville, ON, Canada). Nano cellulous fiber dispersion paste (in water) was purchased from the University of Maine (Orono, ME, USA). Micro-particle aluminum oxide was purchased from SkySpring Nanomaterials Inc. (Houston, TX, USA), and PG022 silica dispersion in water was obtained from Cabot (Alpharetta, GA, USA). Carbon ink (ECI 7001 E&C) was purchased from Loctite (Westlake, OH, USA) and silver ink (5025) was purchased from Dupont (Wilmington, DE, USA). Ag/AgCl ink was purchased from Kayaku Advanced Materials Inc. (Westborough, MA, USA). Polyethylene terephthalate PET (ST 505) films were purchased from Tekra (New Berlin, WI, USA).

### 2.2. Material Preparation and Device Fabrication

Microfluidic devices were fabricated in two steps: material preparation and device printing. Functional and printable materials were formulated and then deposited on PET films using a screen printer or a flexo printer to form the designed structure.

Two types of materials were developed for printing microfluidic devices. Type I contained 16% silica-dispersion fluid (PG022), 28% water, 56% nano cellulous fiber dispersion paste, and 0.7% fumed silica. The first two chemicals were first mixed using a vortex mixer. The third and fourth components were added and mixed in a mechanical mixer sequentially. Type II contained 8.4% silica-dispersion, 14.1% dimethyl sulfoxide (DMSO), 4.7% fumed silica, 43.5% PVA/DMSO solution with 12% PVA, and 29.3% alumina (0.4–10 μm). A vortex mixer was used to mix the first two chemicals, and a mechanical mixer was used to homogenize the mixture with the remaining components.

A patterned stainless-steel stencil was used to print Type I material directly on PET films to construct microfluidic channels, while a patterned 200-mesh stainless steel wire screen was used to print the Type II material. An ASYS EKRA X1-SL semi-automatic screen printer (ASYS Group, Dornstadt, Germany) was used for printing, and the substrate was ST505 polyethylene terephthalate film. The distance between the screen and the substrate was set to approximately 2.4 mm. The devices were printed using a metal squeegee for stencil printing and a 75 Shore A polyurethane squeegee with a 65° attack angle. A pressure of 2 bar was applied to the squeegee, and the print speed was 30 mm/s. The separation speed was set to 0.5 mm/s. A low separation speed was found to increase the print quality. The printed devices were dried at 120 °C for 10–20 min. The Type II mixture was also diluted with DMSO for printing narrower and much thinner nanofluidic channels using flexographic printing. Testacolor 171 from NSM-AG (Zofingen, Switzerland) was used for flexographic printing.

To print electrodes connected to the microfluidic channels, the same screen printer was used. The as-received carbon ink was first printed on PET films using a 200-mesh steel screen, forming the working and counter electrodes. Then, the commercial Ag/AgCl ink was printed on the films using a 200-mesh polyester screen to form reference electrodes. Finally, microfluidic channels were printed over the electrodes after proper alignment. Each layer was dried at 120 °C.

### 2.3. Device Characterization

The structure profile of the printed microfluidic and nanofluidic channels was characterized using a Veeco Dektak 150 profilometer (Veeco, Plainview, NY, USA). The thickness of the screen-printed films was measured using a Model 49-76-00-000 thickness tester (Testing Machines Inc., New Castle, DE, USA). The surface morphology of the printed channels was characterized using a Hitachi scanning electron microscope (SEM). The porosity of the printed Type II materials was estimated by measuring the amount of water absorbed by the printed films.

The fluidic performance of the printed channels was characterized by making video recordings of the transport of water. The addition of very dilute yellow inkjet ink made the water transport much more visible. A measured volume of ink between 0.2 μL and 8 μL, depending on the channel dimension, was applied to the inlets of the channels.

For the application of the printed devices in the colorimetric determination of glucose, the pore surface of the channels was first surface modified using 3-aminopropyltrimethoxysilane. To determine the optimal concentration, 0.5–2% 3-aminopropyltrimethoxysilane solutions were prepared. The hydrolysis reaction was performed in 95% ethanol. The solution was spotted on the detection zone, and the device was heated to 110 °C for 10 min. A 1% 3-aminopropyltrimethoxysilane solution in ethanol was found optimal for subsequent enzyme immobilization. A stock solution was prepared with 5 parts GOx (120 U mL^−1^) and 1 part HRP (30 U mL^−1^) dissolved in a 100 mM phosphate buffer (pH 6). The phosphate buffer solution was prepared with disodium phosphate heptahydrate (0.075 M) and monosodium phosphate monohydrate (0.025 M), adjusted to a pH of 6 with HCl. A second mixture was prepared containing KI (0.6 M) and trehalose (0.3 M) in water. Artificial urine was prepared, and glucose was added to reach the desired concentrations.

In a colorimetric assay, 0.7 μL of the enzyme solution was spotted on each detection zone and dried for 10 min. Then, 0.6 μL of the solution containing the chromogenic agent was spotted on the detection zone and dried for 10 min. The solution spotted in each detection zone had a very small volume that limited its spreading to only cover the zone and a channel section about 1–2 mm from the zone. Then, 9.5 μL of the artificial urine solution was pipetted onto the central zone and allowed to flow to all the detection zones (within 1 min). The samples were placed in a humid environment and allowed to react for 10 min. Afterward, the samples were allowed to dry for an additional 5 min. Color evaluation was performed with a photo scanner of Epson Model Perfection V39 Flatbed Scanner (Epson, Suwa, Japan) using 600 dpi resolution. The color change was quantified by measuring grey scale intensity in ImageJ (2021) relative to the baseline intensity of the 0 mM sample.

For the application of the printed devices in the electrochemical determination of glucose, a Princeton Applied Research PARSTAT 2263 potentiostat (AMETEK Scientific Instrument, Oak Ridge, TN, USA) was used for both cyclic voltammetry and chronoamperometry. In the cyclic voltammetry experiments, 6 µL of 1 mM potassium ferricyanide dissolved in 0.5 M KCl and 0.1 M PBS buffer (pH 7.4) was spotted onto the inlets of the channels of Type II material and allowed to spread through the channels. The experiments were performed between a potential of −0.3 V and 1 V and at scan rates ranging from 25 to 400 mV/s.

In the chronoamperometry experiments, glucose oxidase was used to oxidize glucose to gluconic acid. The oxidation is coupled with the reduction of potassium ferricyanide to potassium ferrocyanide. The ferrocyanide can then be converted back to ferricyanide at a set potential. This conversion causes the current used in chronoamperometry. The glucose samples were prepared in a 0.1 M PBS buffer at pH 7.4 and allowed to mutarotate overnight. Chronoamperometry was performed using a step potential of 400 mV. In total, 1 µL of enzyme solution (800 U mL^−1^ glucose oxidase, 100 mM potassium ferricyanide, 1 M KCl in 0.1 M PBS buffer at pH 7.4) was added directly to the top of the microfluidic channel that covered the working electrode and allowed to dry at room temperature. Then, 6 µL of the glucose sample was added to the channel inlets and allowed to travel through the whole channel (within 1 min). The measurement was performed 10 min after the channel was fully wetted by the sample

## 3. Results and Discussion

### 3.1. Printed Microfluidic Devices

**Figure 1:**
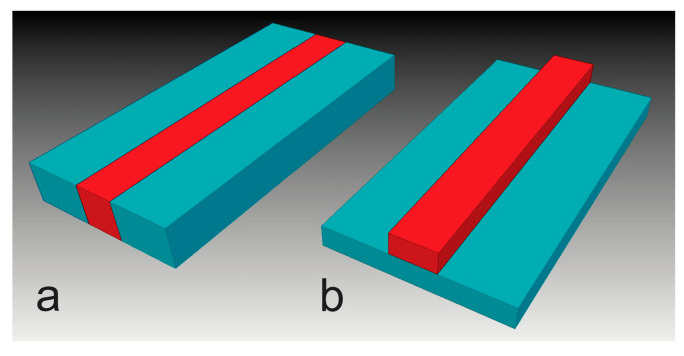
Schematic of a typical paper-based microfluidic channel embedded in paper (**a**), and a free-standing microfluidic channel that sits on a substrate (**b**). The red color section represents the microfluidic channel.

Paper-based microfluidic devices are typically fabricated by injecting a hydrophobic material into porous filter paper to constrain the liquid spreading into a limited space in one dimension. The device channels are thus directly embedded in the paper, as illustrated in Figure 1a with red color, while the injected material forms banks that limit the fluid flow within the channels. The banks in this case act as physical barriers but do not need to withstand the high fluidic pressures that are exerted on the walls of traditional microfluidic channels. The reason is that the fluidic flow in the paper is driven by capillary action, which is controlled by the microstructure and surface chemistry in the paper. This is partly the reason why microfluidic channels can be fabricated by cutting filter paper.

Cut-paper-based microfluidic device channels lack hydrophobic banks and directly expose their side surfaces to air. The high surface energy of cellulose fibers and the interconnected small pores between the fibers hold the liquid within the channels. Our tests showed that when cut-paper-based microfluidic devices are in close contact with a low-energy polymer substrate, an aqueous ink spreads in the paper channels without leaking out. When the devices are in close contact with a high surface energy inorganic substrate, however, an aqueous ink might leak into the substrate during transport. These facts prompted the choice to deposit a specialty porous material directly on a hydrophobic polymer film to build microfluidic devices. A polymer with a strong adhesion to the porous material was selected so that the polymer film could provide a natural support to the devices for mechanical protection and easy handling. The device channels have a free-standing or raised structure on top of the substrate (Figure 1b), in contrast to the more usual embedded channel structure (Figure 1a). Since printing is an additive process and can be used in the mass production of devices at low cost and high precision, printed devices should have clear advantages over cut paper devices that are fabricated in costly one-by-one elimination processes and are difficult to handle directly without backing support.

**Figure 2:**
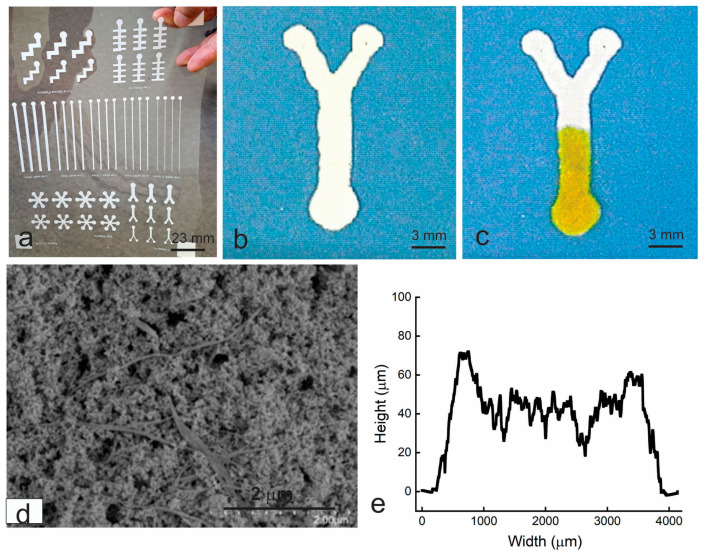
The microfluidic devices printed with type I material on a PET film using stencil printing. (**a**), Multiple devices on a PET film; (**b**), a Y-channel device from (**a**) over blue paper; (**c**), when an aqueous ink is applied onto the inlet of the printed device for 2 s; (**d**), SEM of the printed structure; and (**e**), thickness profile of the Y channel across the main channel.

To fabricate free-standing microfluidic devices using deposition, a paper pulp-like material (Type I) containing cellulous fibers and silica nanoparticles was developed. The material can be cast on PET films to form porous films that have a similar appearance to fine filter papers and can spread water quickly. It can also be deposited on PET films to form narrow lines with stencil printing or syringe dispensing. Figure 2a shows multiple devices printed on a transparent PET film using stencil printing, and Figure 2b,c shows the Y-channel devices on the PET film. Channels with a width of 0.5 mm to 3 mm were printed out. The devices are about 50 μm thick (Figure 2e), and their surface is very porous. Micropores and nanopores (Figure 2d) are formed in the devices with their surface chemistry defined by cellulous fiber and silica surfaces. The printed structure passed the ASTM ink adhesion tape test (ASTM F2252-03 [17]) and has reasonably good adhesion on PET films. When an aqueous ink is applied onto it, the ink travels along the channel quickly, in a fashion very similar to the ink transport in a cut-paper microfluidic device placed on PET films. There is no ink leakage out of the device.

These printed devices demonstrate the feasibility of microfluidic devices that are deposited on top of their supporting substrates, as a free-standing or raised structure, and move fluid by capillary action. Their paper-like appearance and performance are attributed to the interconnected hydrophilic pores formed by cellulose fiber and silica nanoparticles. The cellulose fibers form the backbone network and provide the bonding of the devices with the substrate, while the silica nanoparticles separate the fibers to prevent their aggregation and ensure the formation of interconnected pores. Unfortunately, the material is only compatible with limited deposition processes, such as stencil printing and syringe dispensing. Also, the printed channels are 7% wider than the designed ones in some sections and the channel edges are not straight.

To solve the processing problems encountered with the paper pulp-like material, the concentration of cellulose fibers was substantially reduced and hydroxypropyl methylcellulose was introduced as a binder. This new material shows reasonable dispersion stability and can be screen printed to form a well-defined structure that is bonded firmly to PET films. Water propagates through this medium rapidly, showing a strong capillary effect. In the devices printed with this silica nanoparticle-dominated material, a small amount of cellulose fibers was found to play a vital role in spreading water. With 5% or more cellulose fiber, water spreads rapidly in the printed channels. Otherwise, transport is slow. It is believed that cellulose fibers may connect the nanopores formed by silica nanoparticles and bridge the water transport between pores [16]. Unfortunately, some cellulose fibers may deposit permanently on the screen, and eventually introduce defects in the printed devices after multiple printing runs.

Further improvement required a fiber-free material with better printability. Alumina microparticles were thus introduced to form interconnected hydrophilic pores with silica nanoparticles. Hydrophilic polyvinyl alcohol (PVA) is used as a binder for holding the particles together, bonding the particles to the substrate, and tuning the material viscosity for printing. Interconnected hydrophilic pores can be easily formed in the particle mixture because the packing voids in any solid particle bed are always interconnected, and the surface energies of alumina and silica are high. Both micropores and nanopores are expected in the particle mixture of microparticles and nanoparticles. Based on our experiments [16], the co-existence of the micropores and nanopores is considered essential for achieving the desired capillary flow. Micropores are essential for sufficient volume to flow, but nanopores exert a stronger capillary force that is effective in quickly drawing liquid into the device.

Unfortunately, the high surface energy particles could be covered by PVA, and the pore interconnectivity could be easily interrupted by PVA. In practice, the polymer has to be mixed with the particles in a dissolved state, so that a printable material can be obtained. In this case, PVA could easily migrate onto the particle surface or fill the inter-particle voids, remaining there after the solvent is evaporated. In order to keep this side effect at an acceptable level, the minimum PVA concentration that would give a good material was sought, and this was found to be around 10 wt.%.

**Figure 3:**
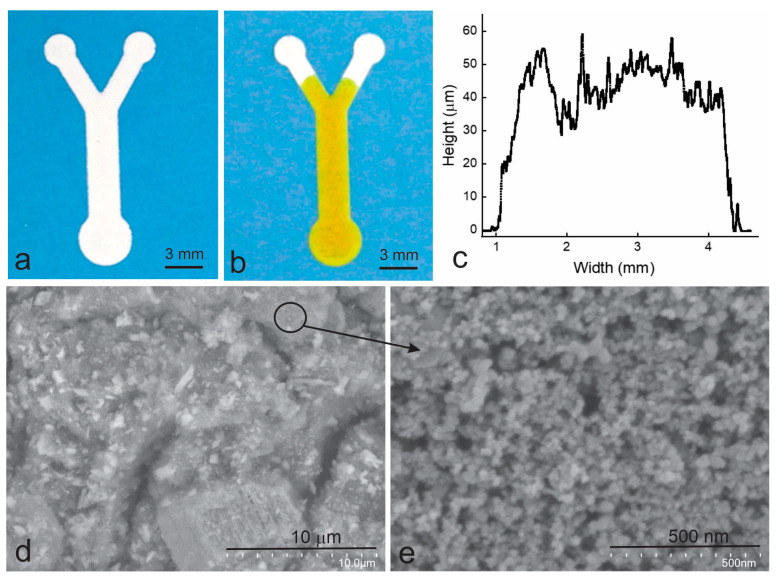
The Y-channel devices printed on PET films using Type II material. (**a**), The printed device; (**b**), 4 s after an aqueous ink is applied to the inlet pad; (**c**), the thickness profile across the main channel; (**d**), SEM of the printed channel; and (**e**), the amplified image of the marked area in (**d**). A screen printer was used to print the devices.

Type II material was obtained from the above development process. It has the appearance of very thick paint and can be printed to form microfluidic structures with sharp boundaries using a screen printer. The structure is mechanically stable after drying and is resistant to surface scratching and substrate bending. Figure 3 shows a Y-channel device obtained by printing Type II material using a screen printer. Its edges are straight and sharp. The structure is about 40 μm thick (Figure 3c) and contains crack-like pores, up to 1 μm wide and 6 μm long (Figure 3d), and small interconnected pores up to 50 nm in diameter (Figure 3e). Its bonding to the supporting PET films is very strong and easily passes the ASTM adhesion tape test. When an aqueous ink is applied to its inlet, the ink travels rapidly (as seen in Figure 3b) in the channel without leaking out, demonstrating excellent capillary flow within the device.

**Figure 4:**
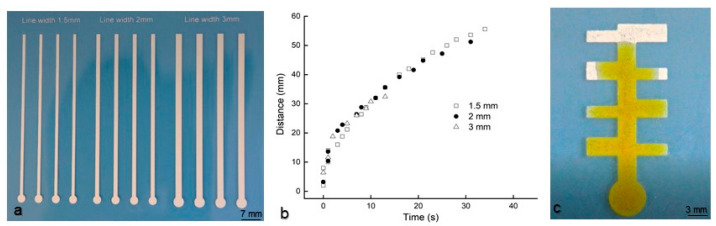
The fluidic transport performance of the microfluidic devices that were printed with Type II material on a PET film. (**a**), Straight channels of varying width; (**b**), flow kinetics of aqueous ink in the straight channel devices (from (**a**)) labeled with the channel width; and (**c**), 6 s after an aqueous ink applied onto the inlet of a printed tree-shaped device. A screen printer was used to print the devices.

Figure 4a shows some straight-channel devices printed with Type II material, and Figure 4b shows the ink flow kinetics within the channels with different widths. The aqueous ink flows rapidly in the section close to the inlet and then slows down as it travels further. The flow at the beginning is complicated, as part of the applied ink was observed moving rapidly on the channel surface. After passing a certain distance, the ink only flows within the channels and the flow velocity gradually reduces with time from 1.4 mm/s to 0.8 mm/s. While this velocity is higher than the flow velocity in typical paper-based microfluidic channels, the flow within the printed channels has similar characteristics to the flow in typical paper-based microfluidic channels [18,19,20]. This type of one-dimensional capillary flow was studied in paper channels using the Washburn equation and other models [19].

For channel widths from 1.5 mm to 3 mm, the flow velocity was almost independent of the channel width (Figure 4b). To confirm this result, a device that includes channel branches of different widths was printed and tested. As seen from the image in Figure 4c, the aqueous ink flows the same distance in the wide and narrow channels. The main reason for this behavior is that the printed channels are raised from the substrate with no side walls. The channels are in contact with air, which does not affect the capillary-driven fluidic flow within the channel. This is different from the situation in some typical paper microfluidic channels that have solid barriers or banks. The solid barriers created by filling paper pores with wax or other hydrophobic materials might cause drag, resulting in a channel width-dependent flow [19].

The overall porosity in the printed devices was measured at around 24%, and the typical thickness of the devices was about 40 μm. As such, the required sample volume for filling the pores in the printed devices is about 0.010 μL/mm^2^. In comparison, the sample volume of typical paper-based microfluidic devices made from filter papers with over 60% porosity and 180 μm thickness [21,22] is over 0.108 μL/mm^2^. Hence, for the same 2D area, a printed microfluidic device needs only about 10% of the sample required by a paper-based device to fill all the involved pores. Furthermore, this small sample volume can be greatly reduced by sightly diluting the same material and printing functional devices using a flexo printer. Printed devices, about 10 μm thick, were obtained in this way in our lab, resulting in a required sample volume of about 0.003 μL/mm^2^, close to the range of nanofluidics.

Both screen printing and flexo printing are suitable for high-volume and low-cost device fabrication, as demonstrated in the printable electronics industry. Quality and multiple devices can be simultaneously printed on a single substrate (as seen in Figure 2a) using these methods, which implies that printed microfluidic devices may benefit from this advantage. Furthermore, as microfluidic devices are in millimeter or submillimeter dimensions, the sheet-to-sheet variation in device structure and device position during a print run is negligible. This allows easy high-volume device printing and easy processes for subsequent reagent doping, device sealing, and cutting. In addition, the devices are directly printed on PET films that naturally provide good mechanical support to the devices. This eliminates a post-process for attaching backing films as required in fabricating paper-based microfluidic devices, and thus further keeps the device fabrication cost low.

### 3.2. Applications of Printed Microfluidic Devices in Glucose Sensing

The printed microfluidic devices were first applied in colorimetric detection of glucose, as this method was successfully demonstrated with paper-based microfluidic devices [23,24,25]. Since the anchoring of chromogenic agents is a crucial step to ensure reliable analytical information from colorimetric measurements [26], our first step was to develop the anchoring method for the printed devices. For paper-based microfluidic devices, it was reported that modification with chitosan can decrease the color gradient [27]. Unfortunately, chitosan, which is ideal for enzyme immobilization onto cellulose fibers, was found to be ineffective in improving the color uniformity of the printed devices. The reason is that the particles used in the printed devices do not have a cellulose-type structure to interact strongly with chitosan.

**Figure 5:**
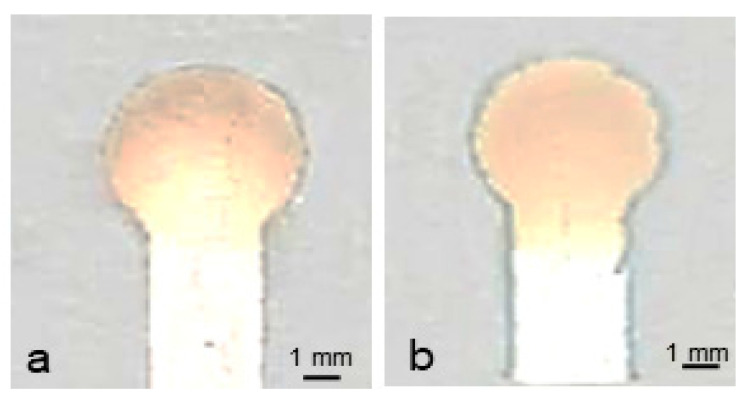
Effect of surface modification on color uniformity. An enzyme solution was spotted on the detection zone and 5 mM glucose solution was introduced to the device channels which were: (**a**), as-printed and (**b**), surface-modified with 1% 3-aminopropyl trimethoxysilane solution. Type II material was used to print the devices with a screen printer.

Since the printed devices are composed of alumina and silica particles and the pores (Figure 3d,e) in the devices are interparticle voids, 3-aminopropyltrimethoxysilane was chosen for surface modification. This chemical is widely used in surface modification for biosensing [28], as it contains three hydrolysable substituents to react with up to three OH groups on the alumina and silica particles and an amine group that can interact with an enzyme. The surface-modified devices were compared with the unmodified devices in subsequent glucose sensing. The former ones (Figure 5b) were seen to give higher color uniformity than the latter ones (Figure 5a). This surface modification was thus used for all subsequent tests.

**Figure 6:**
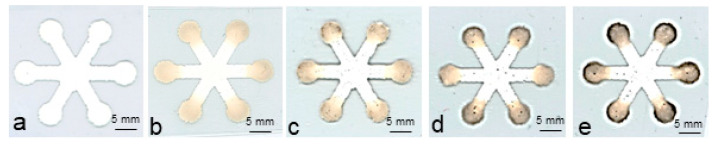
Images of the printed microfluidic devices tested with artificial urine solutions with varying concentrations of glucose: (**a**), 0 mM; (**b**), 5 mM; (**c**), 10 mM; (**d**), 15 mM; and (**e**), 25 mM. Type II material was used to print the devices using a screen printer.

**Figure 7:**
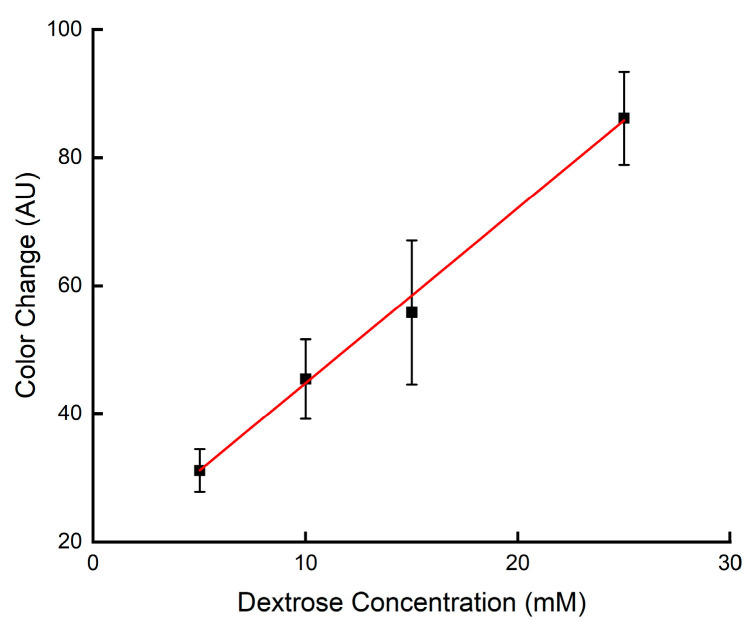
Analytical curve for glucose assay on the printed microfluidic devices using KI as the colorimetric indicator. The solid line represents a linear fit to the data with the regression equation: y = 2.7257x + 17.128 (R^2^ = 0.9958).

With the pore surface modified, the colorimetric method used for paper-based microfluidic sensors [27,29] was used in glucose sensing for the printed channels. In this application, star-shaped devices, each containing six identical channels and six detection zones, were printed and surface-modified. Firstly, enzyme solution was spotted on each detection zone and dried, followed by applying the solution containing the chromogenic agent. Then, an artificial urine solution, prepared using a reported method [30], was pipetted onto the central zone and allowed to flow to the six detection zones of each printed device, where the reaction occurred. Figure 6 shows images of the devices tested with artificial urine solutions with various glucose concentrations. Each detection zone became darker after the reaction, and the color intensity increased with the glucose concentration. The scanned images in all the detection zones in each device were quantified by measuring the greyscale intensity with Image J software to obtain the results in Figure 7. A large standard deviation, similar to that reported in some paper-based devices [29], was observed in this testing. It could be reduced by choosing a more suitable color indicator and using a better image capture tool.

The limit of detection (LOD) of the devices was calculated based on the ratio between 3 times the standard deviation for the blank sample and the slope of the analytical curve [27]. A value of 0.38 mM was obtained for the printed devices. The LOD of comparable microfluidic devices fabricated from cellulose paper, using KI as a colorimetric indicator, is in the range of 0.1 mM to 2.8 mM [3,27,29,31]. The LOD of the printed microfluidic devices falls within this range, indicating that their performance is comparable to paper-based microfluidic devices with a similar device structure using the same testing method.

The above tests also showed that the printed microfluidic devices can handle small sample volumes. Spotting 9.5 μL of an artificial urine sample at the center of the device is sufficient to supply all six detection zones of the star-shaped device. This is much lower than the 70 μL required by the devices fabricated from filter paper-based microfluidic devices with a similar device structure [27]. This confirms the earlier analysis that printed microfluidic devices can handle much smaller sample volumes per device area than paper-based microfluidic devices.

**Figure 8:**
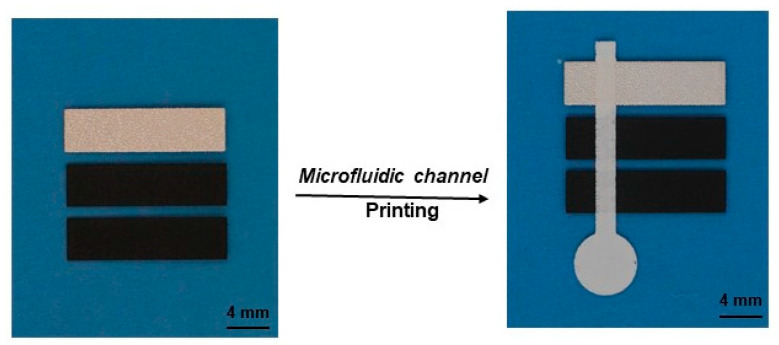
Printed electrodes and their integration with printed microfluidic channels. Carbon electrodes (working and counter) and Ag/AgCl electrodes (reference) were printed on a PET film before the microfluidic channels were printed over top. Screen printing was used for the printing, and Type II material was used for the microfluidic channels.

In addition to the application in colorimetric glucose detection, the printed microfluidic devices were also applied in electrochemical glucose detection, by following the methods successfully demonstrated with paper-based microfluidic channels [32,33]. To fabricate the electrochemical devices, the working and counter electrodes were first printed on PET films using screen printing with commercial carbon ink, followed by the printing of reference electrodes using commercial Ag/AgCl ink. Then, microfluidic channels were printed over the electrodes using Type II material. Figure 8 shows this integration of the electrodes with microfluidic channels. To enable a direct comparison, the device structure was adopted from a reported paper-based microfluidic device that was fabricated using printing electrodes on paper [33].

**Figure 9:**
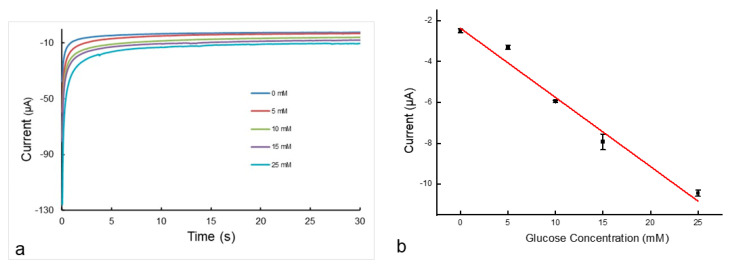
(**a**), Representative chronoamperometric curves for glucose concentrations ranging from 0 mM to 25 mM. (**b**), Calibration plot of current as a function of the glucose concentration. The solid line represents a linear fit to the data with regression equation: y = −0.3369x − 2.317 (R^2^ = 0.9765).

The printed-device performance in cyclic voltammetry was evaluated first to confirm the reversible redox reaction of potassium ferricyanide and diffusion-controlled kinetics, as reported in paper-based devices [32,33]. Then, the devices were tested for chronoamperometry for glucose sensing by following a reported method [32,33]. As explained above, glucose oxidase produces gluconic acid with concomitant reduction of potassium ferricyanide to potassium ferrocyanide. Electrochemical oxidation back to ferricyanide gives the current that can be measured using chronoamperometry. The current over time was measured for various concentrations of glucose, as shown in Figure 9a. The observed current after 30 s was used to create a calibration curve, as seen in Figure 9b. The calibration curve shows that the current is linearly proportional to the glucose concentration when the concentration is between 0 and 25 mM. A limit of detection (LOD) of 0.25 mM was calculated using the method reported in [27]. This LOD value is similar to the value of 0.21–0.35 mM obtained from the electrochemical detection of paper-based microfluidic devices [32,33,34]. It shows that the performance of the printed devices is comparable to paper-based devices with similar structures when using the same test method.

The above tests again showed that the printed microfluidic devices can handle small sample volumes: 6 μL of the testing solution spotted on the device is sufficient for the detection. This is much smaller than the 100 μL required by the devices fabricated from filter paper-based microfluidic devices with an identical device structure [33].

Printing electrodes on flat PET films and integrating them with printed microfluidic channels is straightforward. First, conductive carbon ink and Ag/AgCl inks can be easily printed, and inks with a wide range of sheet resistances are commercially available. Second, fine alignment can be easily achieved to ensure precise integration of each involved component. For paper-based microfluidic devices, the typical filter paper used for microfluidic devices is characterized by extremely rough surfaces. It is difficult to print electrodes on the surfaces to achieve good continuity. Thick electrodes made using multiple conventional printing passes or using non-standard printing techniques are normally required to obtain the electrical conduction required for testing [33,34,35]. For instance, 100 μm thick electrodes were used for paper-based microfluidic devices [33], in comparison with 37 μm for the printed carbon electrodes in this work. It is also difficult to precisely integrate printed electrodes into paper-based microfluidic channels because it is difficult to create high-resolution alignment marks on filter paper.

As printed microfluidic channels can be directly fabricated using conventional printing processes, the integration of microfluidic channels with other components on the same substrate can be therefore easily achieved, as demonstrated in this work. With this, it is possible to fabricate quality integrated devices in volume at low cost. In our recent effort in electrochemical glucose detection, for instance, an LOD of 0.08 mM was easily achieved by simply printing lower resistance electrodes in the above devices.

## 4. Conclusions

Capillary-driven microfluidic devices with free-standing structures were directly printed on PET films, using our specifically developed materials. Devices that contain hydrophilic interconnected pore systems can be produced either by screen printing or flexo printing using the materials. The printed devices have sufficient adhesion with the PET substrate and good mechanical stability, and their upper and side surfaces are exposed to air. Water and aqueous solutions flow in the printed devices in the same fashion as they do in paper-based microfluidic devices. The printed microfluidic devices can handle a sample of about 10% of that required by typical filter paper-based microfluidic devices due to their lower porosity and smaller thickness.

The printed microfluidic devices were preliminarily applied to colorimetric detection of glucose and electrochemical detection of glucose using reported device structures and testing methods. The obtained LODs of the printed microfluidic devices are similar to the ones reported for the corresponding paper-based microfluidic devices with similar device structures using the same test method. The printed microfluidic devices can provide similar sensing performance to paper-based ones and can be treated as an alternative capillary microfluidic platform.

## Data Availability

The data are available upon reasonable request from the corresponding author.
